# Exploring the relationship between BMI and cognitive function in older adults: The mediating role of recreational physical activity

**DOI:** 10.1097/MD.0000000000048578

**Published:** 2026-05-01

**Authors:** Zheng Zhang, Juan Li

**Affiliations:** a Department of Sports Science, Yancheng Institute of Technology, Yancheng, China; b Department of Sports Science, Hanyang University, AnSan, South Korea.

**Keywords:** BMI, cognitive function, cross-sectional study, NHANES, recreational physical activity

## Abstract

This study investigates the associations among body mass index (BMI), recreational physical activity, and cognitive function in older adults, and examines whether recreational physical activity mediates the association between BMI and cognitive function. This cross-sectional study used data from 1213 adults aged 60 years and older from the 2011 to 2014 National Health and Nutrition Examination Survey. Multiple linear regression analyses were performed to evaluate the associations among BMI, recreational physical activity, and cognitive function. Mediation analysis was conducted to assess the mediating effect of recreational physical activity. Models were adjusted for age, sex, ethnicity, and smoking status. BMI was negatively associated with cognitive function in the fully adjusted model (β = −0.0074, 95% confidence interval [CI]: −0.0144 to −0.0004, *P* = .038). Recreational physical activity was positively associated with cognitive function (β = 0.0002, 95% CI: 0.0000–0.0003, *P* < .005), whereas BMI was negatively associated with recreational physical activity (β = −9.08, 95% CI: −12.80 to −5.36, *P* < .001). Recreational physical activity partially mediated the association between BMI and cognitive function, accounting for 16.95% of the total effect (*P* = .0320). Among older adults, higher BMI was associated with lower cognitive function, and recreational physical activity partially mediated this association.

## 1. Introduction

Cognitive function refers to the brain’s core ability to process information, encompassing attention, memory, language, executive function, and problem-solving.^[1]^ These skills are fundamental to maintaining independence in daily life and facilitating social participation.^[2,3]^ They underpin an individual’s capacity to learn and adapt to their environment, making the proper functioning of cognitive processes essential for a high quality of life.^[4]^ However, cognitive decline significantly impacts the health and well-being of older adults. Although the natural decline in cognitive function with age is inevitable, the degree and rate of decline vary widely across individuals.^[5]^ Research has identified cognitive decline as an early marker of neurodegenerative diseases, such as Alzheimer disease, and it is strongly associated with other health issues, including depression and social isolation.^[6–8]^ Understanding and preserving cognitive function has thus become a central focus in aging research.

Body mass index (BMI) is a widely used measure to assess body fat and its potential health implications, serving as a simple and reliable indicator of an individual’s weight relative to height.^[9]^ BMI is closely linked to numerous health conditions, including obesity, cardiovascular disease, and metabolic syndrome.^[10]^ It also provides a valuable basis for studying the relationship between weight fluctuations and other health outcomes. Elevated BMI is known to influence bodily functions through various mechanisms, including chronic inflammation, hormonal dysregulation, and increased metabolic burden.^[11,12]^ At the neurological level, high BMI has been associated with alterations in brain structure and function, such as reduced gray matter volume, impaired white matter integrity, and decreased neuroplasticity.^[13]^ Moreover, its connection to mental health disorders, such as depression and anxiety, highlights the systemic impact of BMI on overall health.^[14]^ Although elevated BMI is commonly viewed as a health risk marker, its role can vary based on population, age, and other contextual factors. In older adults, both high and low BMI have been linked to adverse health outcomes, resulting in a “U-shaped” association.^[15]^ Understanding the mechanisms by which BMI influences specific health domains, such as cognitive function, is essential for designing effective interventions.

Recreational physical activity is a key lifestyle factor for promoting overall health and is recognized for its role in preventing and mitigating chronic diseases.^[16]^ Evidence suggests that physical activity enhances both physical and cognitive health through mechanisms such as improved cardiorespiratory fitness, reduced systemic inflammation, enhanced neuroplasticity, and increased cerebral blood flow.^[17]^ Particularly in older populations, physical activity is considered a crucial intervention for slowing cognitive decline.^[18]^ Furthermore, recreational physical activity may mediate the relationship between BMI and cognitive function.^[19]^ Higher BMI is often associated with lower levels of physical activity, which may diminish the health benefits conferred by physical activity.^[20]^ Conversely, recreational physical activity can mitigate some of the adverse effects of elevated BMI by improving metabolic status and reducing psychological stress, thereby alleviating its negative impact on cognitive function.

Although the relationships among BMI, physical activity, and cognitive function have been studied extensively, few investigations have systematically explored the mechanisms by which recreational physical activity serves as a mediator. This study aims to elucidate the mediating role of recreational physical activity in the relationship between BMI and cognitive function using quantitative data. By doing so, it provides novel insights into the complex interactions among these factors and establishes a scientific foundation for developing effective strategies to enhance cognitive health in older populations.

## 2. Methods

### 2.1. Study population

**Figure 1.**
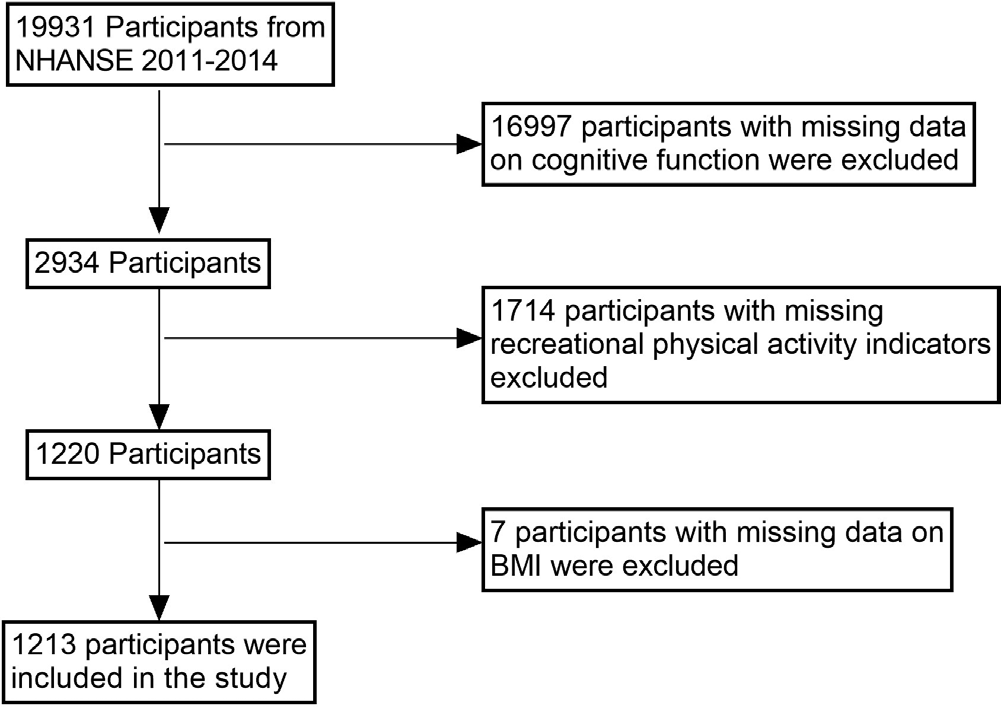
Flowchart. NHANES = National Health and Nutrition Examination Survey.

This study utilized data from the 2011 to 2014 National Health and Nutrition Examination Survey (NHANES), initially including 19,931 participants. Following the inclusion criteria, individuals with missing data on cognitive function (n = 16,997), recreational physical activity indicators (n = 1714), and BMI (n = 7) were excluded sequentially, resulting in a final sample of 1213 participants aged ≥60 years. All included participants had complete data on BMI, cognitive function, and recreational physical activity. The sample was selected using a stratified multistage probability sampling design to ensure national representativeness. Ethical approval for the study protocol was obtained, and all participants provided written informed consent. A detailed flowchart of the sample selection process is presented in Figure 1. This rigorously selected sample offers a robust foundation for investigating the relationship between BMI and cognitive function and the mediating role of recreational physical activity in older adults.

### 2.2. Measurement of BMI

In the NHANES, BMI was measured by professionally trained medical technicians at the Mobile Examination Center. Weight and height were obtained using high-precision digital scales and fixed upright stadiometers, respectively, ensuring data accuracy and consistency. BMI was calculated as weight in kilograms divided by height in meters squared (kg/m^2^).

### 2.3. Recreational physical activities assessment

Recreational physical activity in the NHANES was assessed using a questionnaire. Participants were asked 2 key questions: “On a typical day, how much time do you (or the respondent) spend engaging in vigorous sport, fitness, or recreational activities?” and “On a typical day, how much time do you (or the respondent) spend engaging in moderate sport, fitness, or recreational activities?” Responses were recorded in minutes.

To quantify recreational physical activity, metabolic equivalents (METs) were used to calculate total daily activity. The duration of moderate-intensity activity was multiplied by 4 METs, while the duration of vigorous-intensity activity was multiplied by 8 METs.^[21,22]^ The sum of these 2 values represented the total level of recreational physical activity (MET-min/day). This methodology adheres to internationally recognized standards for classifying activity intensity, providing a robust quantitative framework for examining the mediating role of recreational physical activity in the relationship between BMI and cognitive function.

### 2.4. Measurement of cognitive function

Cognitive function was assessed in study participants using data from the NHANES database (2011–2014). Cognitive performance was evaluated across 4 domains using the following standardized tests:

1. Animal fluency test
2. The animal fluency test is a verbal fluency assessment in which participants name as many items as possible within a specified category (e.g., animals) during a fixed time period. This test evaluates semantic memory and executive function.^[23]^
3. Digit symbol substitution test
4. The digit symbol substitution test, a component of the Wechsler Adult Intelligence Scale, measures processing speed, attention, and executive function. Participants complete a digit-symbol matching task within a designated time limit.^[24]^
5. Immediate recall
6. Immediate recall evaluates short-term memory by requiring participants to immediately recall a list of words presented to them moments earlier.^[25]^
7. Delayed recall
8. Delayed recall assesses long-term memory by asking participants to recall a previously presented word list after a delay period.^[25]^

To standardize and facilitate comparisons across these cognitive tests, *Z*-scores were calculated for each measure. These standardized scores enable the integration of diverse cognitive domains into a unified framework for analysis, ensuring comparability of results across different metrics.^[26]^ The standardized score (*Z*_*i*_) for the *i*th participant was calculated using the formula:


$$\begin{array}{l} Z_{i}=\frac{X_{i}-\mu }{\sigma } \end{array}$$

where

- *Z*_*i*_ is the standardized score for the *i*th participant,
- *X*_*i*_ is the raw score of the participant,
- μ represents the overall mean score for the test, and
- σ denotes the overall standard deviation for the test.

This standardization process allows for direct comparison across cognitive tests by accounting for variations in scale and distribution.

A composite cognitive function score was calculated for each participant by averaging their standardized scores across the 4 cognitive tests. The formula for the composite score is given as:


$$\begin{array}{l} Cognitivefunctionscore=\frac{Z_{AFT}+Z_{DSST}+Z_{IR}+Z_{DR}}{4} \end{array}$$

This composite scoring method provides a comprehensive assessment of overall cognitive ability by integrating multiple dimensions of cognitive function. It effectively facilitates the exploration of the relationship between BMI and cognitive function in older adults, as well as the mediating role of recreational physical activity, quantified as METs, in this association.

### 2.5. Assessment of covariates

In this study, all multiple regression analyses were adjusted for key covariates, including age, sex, race/ethnicity, and smoking status, to mitigate potential confounding effects. Age was treated as a continuous variable, while sex (male vs female), race/ethnicity (non-Hispanic White, non-Hispanic Black, Mexican American, Other Hispanic, and Other race–including multiracial), and smoking status (never smoked, former smoker, current smoker) were included as categorical variables. These covariates were selected based on their potential influence on BMI, recreational physical activity measured as METs, and cognitive functioning. Adjusting for these factors ensured the robustness of regression model estimates and provided a more precise foundation for exploring the relationships among these variables.

### 2.6. Statistical analysis

Statistical analyses were conducted using EmpowerStats software (version 4.2; X&Y Solutions, Inc., Boston). Because NHANES uses a complex, multistage probability sampling design, all analyses incorporated appropriate sample weights to obtain nationally representative estimates. Descriptive statistics were used to summarize the characteristics of the study population, including means and standard deviations for BMI, recreational physical activity, and cognitive function, as well as the distributions of covariates such as age, sex, ethnicity, and smoking status. Correlation analyses were performed to examine the associations among BMI, recreational physical activity, and cognitive function. Multiple linear regression models were then constructed to evaluate the associations of BMI and recreational physical activity with cognitive function, with progressive adjustment for age, sex, ethnicity, and smoking status. In addition, restricted cubic spline regression was used to examine the potential nonlinear association between BMI and cognitive function. All statistical tests were 2-sided, and *P* < .05 was considered statistically significant.

**Figure 2.**
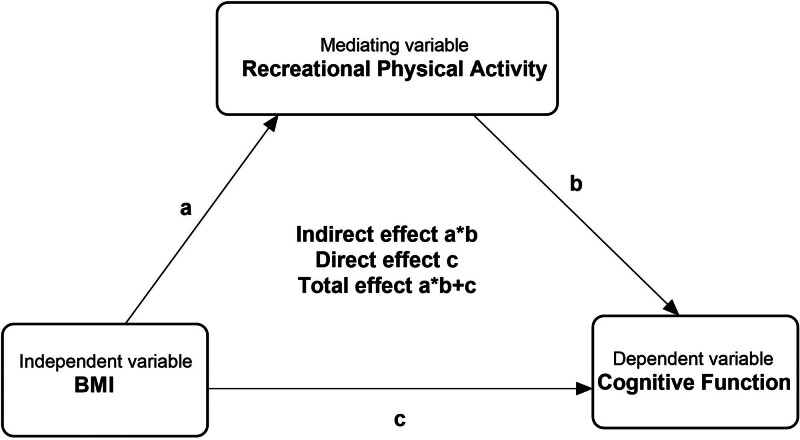
Mediation effects of recreational physical activity on BMI and cognitive function scores. BMI = body mass index.

To further examine whether recreational physical activity mediated the association between BMI and cognitive function, a mediation model was constructed (Fig. 2). In this model, path a represented the association between BMI and recreational physical activity, path b represented the association between recreational physical activity and cognitive function, and path c represented the direct association between BMI and cognitive function. The indirect effect was estimated as the product of path a and path b (a × b), and the total effect was calculated as the sum of the indirect and direct effects (a × b + c). The proportion mediated was calculated as the ratio of the indirect effect to the total effect.

To assess the stability of the mediation results, 1000 bootstrap samples were used to estimate bias-corrected confidence intervals (CIs) for the indirect effect.

## 3. Results

### 3.1. Participants’ characteristics

A total of 1213 participants were included in the study, with a mean age of 68.92 ± 6.64 years. The mean metabolic equivalent of recreational physical activity (MET) was 349.14 ± 361.48, and the mean BMI was 28.23 ± 5.52 kg/m^2^. Cognitive function scores, standardized as *z*-scores, had a mean value of 0.00 ± 0.78. The gender distribution was balanced, with 50.95% (n = 618) male and 49.05% (n = 595) female participants. Racial and ethnic composition included 47.40% (n = 575) non-Hispanic White, 22.84% (n = 277) non-Hispanic Black, 8.57% (n = 104) Mexican American, 8.99% (n = 109) other Hispanic, and 12.20% (n = 148) Other or multiracial. Regarding marital status, the majority of participants were married (57.71%, n = 700), while the remainder were widowed (16.98%, n = 206), divorced (14.76%, n = 179), separated (1.98%, n = 24), never married (5.19%, n = 63), or living with a partner (3.05%, n = 37). Smoking behavior varied, with 51.85% (n = 629) identified as never smokers, 39.57% (n = 480) as former smokers, and 8.57% (n = 104) as current smokers. Alcohol consumption showed that 77.91% (n = 945) of participants were current drinkers, while 13.27% (n = 161) had never consumed alcohol, and 8.82% (n = 107) were former drinkers. Educational attainment revealed a diverse range of backgrounds: 31.90% (n = 387) had a college degree or higher, 29.76% (n = 361) had some college or an associate degree, 21.19% (n = 257) had completed high school or an equivalent qualification, 9.48% (n = 115) had completed grades 9 to 11, and 7.58% (n = 92) had not completed grade 9.

These demographic characteristics, summarized in Table 1, provide a diverse and representative sample, supporting a comprehensive analysis of the relationships among BMI, recreational physical activity, and cognitive function in older adults.

**Table 1:** Characteristics of study population.

	Total (N = 1213)
Age (yr)	68.92 ± 6.64
MET	349.14 ± 361.48
BMI (kg/m^2^)	28.23 ± 5.52
Cognitive function (score)	0.00 ± 0.78
Gender	
Male	618 (50.95%)
Female	595 (49.05%)
Race	
Mexican American	104 (8.57%)
Other Hispanic	109 (8.99%)
Non-Hispanic White	575 (47.40%)
Non-Hispanic Black	277 (22.84%)
Other race–including multiracial	148 (12.20%)
Marital status	
Married	700 (57.71%)
Widowed	206 (16.98%)
Divorced	179 (14.76%)
Separated	24 (1.98%)
Never married	63 (5.19%)
Living with partner	37 (3.05%)
Smoking status	
Never smoking	629 (51.85%)
Former smoking	480 (39.57%)
Current smoking	104 (8.57%)
Alcohol status	
Never drinking	161 (13.27%)
Former drinker	107 (8.82%)
Current drinker	945 (77.91%)
Education level	
<9th grade	92 (7.58%)
9–11th grade	115 (9.48%)
High school graduate/GED or equivalent	257 (21.19%)
Some college or AA degree	361 (29.76%)
College graduate or above	387 (31.90%)

BMI = body mass index, GED = General Educational Development, MET = metabolic equivalent.

### 3.2. Associations between BMI and cognitive function scores

A significant negative association between BMI and cognitive function scores was observed in the fully adjusted model. Each unit increase in BMI was associated with a significant reduction in cognitive function scores (β = −0.0074, 95% CI: −0.0144 to −0.0004, *P* = .038) after adjusting for age, sex, ethnicity, and smoking status (Model 3).

**Figure 3.**
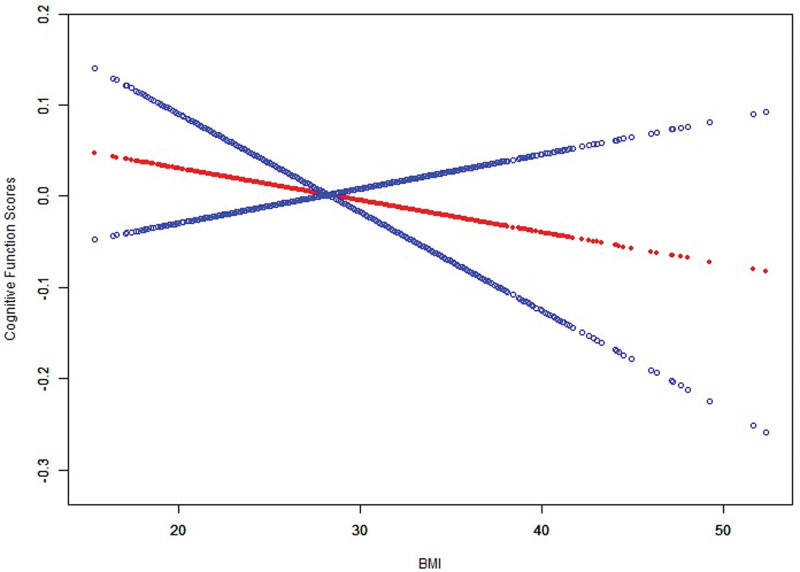
Association between BMI and cognitive function scores with smoothed curve fitting. BMI = body mass index.

When BMI was categorized into tertiles, cognitive function scores were significantly lower in the second (Q2) and highest (Q3) tertiles compared with the lowest tertile (Q1, reference group). In Model 3, cognitive scores were reduced by 0.10 (β = −0.10, 95% CI: −0.19 to −0.01, *P* = .029) in the Q2 group and by 0.12 (β = −0.12, 95% CI: −0.21 to −0.02, *P* = .014) in the Q3 group. These findings indicate that higher BMI levels are associated with poorer cognitive function, particularly among individuals in the upper BMI tertiles, as summarized in Table 2. Smoothed curve fitting further corroborated these results, revealing a declining trend in cognitive function scores with increasing BMI. This visualized trend suggests a potential dose-response relationship between BMI and cognitive function, as shown in Figure 3.

**Table 2:** Associations between BMI and cognitive function scores.

	Model 1		Model 2		Model 3	
	β (95% CI)	*P*	β (95% CI)	*P*	β (95% CI)	*P*
CFS, per-unit increase	−0.0005 (−0.0083 to 0.0074)	.908	−0.0071 (−0.0141 to −0.0002)	.044	−0.0074 (−0.0144 to −0.0004)	.038
BMI levels						
Q1	Ref	–	Ref	–	Ref	–
Q2	−0.05 (−0.16 to 0.05)	.321	−0.09 (−0.18 to −0.00)	.034	−0.10 (−0.19 to −0.01)	.029
Q3	−0.05 (−0.15 to 0.06)	.380	−0.11 (−0.21 to −0.02)	.017	−0.12 (−0.21 to −0.02)	.014

Model 1: unadjusted model.

Model 2: adjusted for age, sex, race, and ethnicity.

Model 3: further adjusted for smoking status in addition to Model 2.

BMI = body mass index, CFS = cognitive function scores.

### 3.3. Associations between BMI and recreational physical activities (MET)

A significant negative correlation between BMI and recreational physical activity (MET) was observed in the fully adjusted model (Model 3). After controlling for age, sex, race, and smoking status, each unit increase in BMI was associated with a significant reduction in the metabolic equivalent of recreational physical activity (β = −9.08, 95% CI: −12.80 to −5.36, *P* < .001). This finding suggests that higher BMI levels are linked to lower participation in recreational physical activity.

In analyses stratified by BMI quartiles, recreational physical activity levels were significantly lower in the highest BMI quartile (Q3) compared with the lowest quartile (Q1, reference group; β = −112.88, 95% CI: −163.14 to −62.62, *P* < .001). However, no significant difference was observed between the second quartile (Q2) and the reference group (β = −7.49, 95% CI: −55.23 to 40.26, *P* = .759), as shown in Table 3. These results highlight the inhibitory effect of elevated BMI on engagement in recreational physical activity, particularly in individuals with the highest BMI levels.

**Table 3:** Associations between BMI and MET.

	Model 1		Model 2		Model 3	
	β (95% CI)	*P*	β (95% CI)	*P*	β (95% CI)	*P*
MET, per-unit increase	−8.5107 (−12.1272 to −4.8943)	<.001	−8.9412 (−12.6174 to −5.2651)	<.001	−9.0819 (−12.8012 to −5.3626)	<.001
BMI levels						
Q1	Ref	–	Ref	–	Ref	–
Q2	5.74 (−41.70 to 53.18)	.813	−7.68 (−55.27 to 39.92)	.752	−7.49 (−55.23 to 40.26)	.759
Q3	−103.42 (−152.56 to −54.27)	<.001	−111.83 (−161.68 to −61.98)	<.001	−112.88 (−163.14 to −62.62)	<.001

Model 1: unadjusted model.

Model 2: adjusted for age, sex, race, and ethnicity.

Model 3: further adjusted for smoking status in addition to Model 2.

BMI = body mass index, MET = metabolic equivalent.

**Figure 4.**
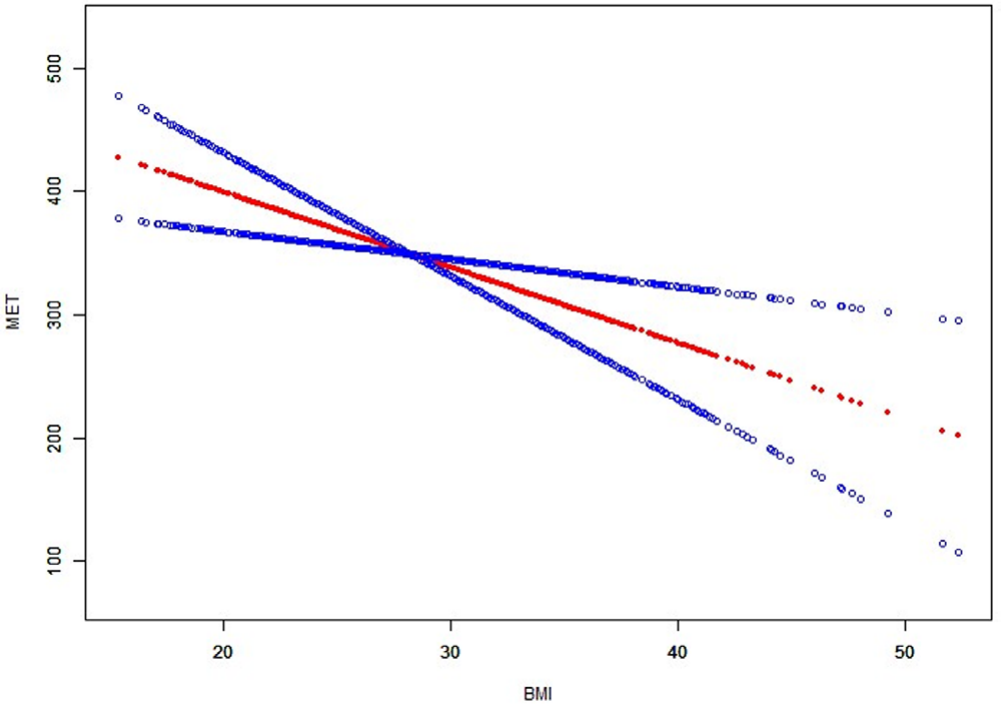
Association between BMI and MET with smoothed curve fitting. BMI = body mass index, MET = metabolic equivalent.

Smoothed curve fitting (Fig. 4) further revealed a nonlinear relationship between BMI and MET levels. MET levels demonstrated a consistent decreasing trend with increasing BMI, with the decline being particularly pronounced in the higher BMI range. These findings suggest that elevated BMI may negatively affect physical activity levels by limiting an individual’s ability or willingness to engage in recreational physical activities.

### 3.4. Associations between recreational physical activities (MET) and cognitive function scores

A significant positive correlation between recreational physical activity (MET) and cognitive function scores was observed in the fully adjusted model (Model 3). After controlling for age, gender, ethnicity, and smoking status, each unit increase in MET was significantly associated with an increase in cognitive function scores (β = 0.0002, 95% CI: 0.0000–0.0003, *P* < .005). This finding suggests that higher levels of recreational physical activity may play a beneficial role in enhancing cognitive function.

Subgroup analyses revealed that individuals in the highest quartile of MET (Q3) had significantly higher cognitive function scores compared with those in the lowest quartile (Q1, reference group; β = 0.20, 95% CI: 0.10–0.29, *P* < .0001). However, no statistically significant difference was observed between the second quartile (Q2) and the reference group (β = 0.07, 95% CI: −0.03 to 0.16, *P* = .1741), as shown in Table 4. These results highlight the potential cognitive benefits of engaging in higher levels of recreational physical activity.

**Table 4:** Associations between MET and cognitive function scores.

	Model 1		Model 2		Model 3	
	β (95% CI)	*P*	β (95% CI)	*P*	β (95% CI)	*P*
CFS, per-unit increase	0.0002 (0.0001–0.0003)	<.005	0.0002 (0.0000–0.0003)	<.05	0.0002 (0.0000–0.0003)	<.005
MET levels						
Q1	Ref	–	Ref	–	Ref	–
Q2	0.17 (0.06–0.28)	.0020	0.07 (−0.03 to 0.16)	.1636	0.07 (−0.03 to 0.16)	.1741
Q3	0.32 (0.21–0.42)	<.0001	0.20 (0.10–0.29)	<.0001	0.20 (0.10–0.29)	<.0001

Model 1: unadjusted model.

Model 2: adjusted for age, sex, race, and ethnicity.

Model 3: further adjusted for smoking status in addition to Model 2.

CFS = cognitive function scores, MET = metabolic equivalent.

**Figure 5.**
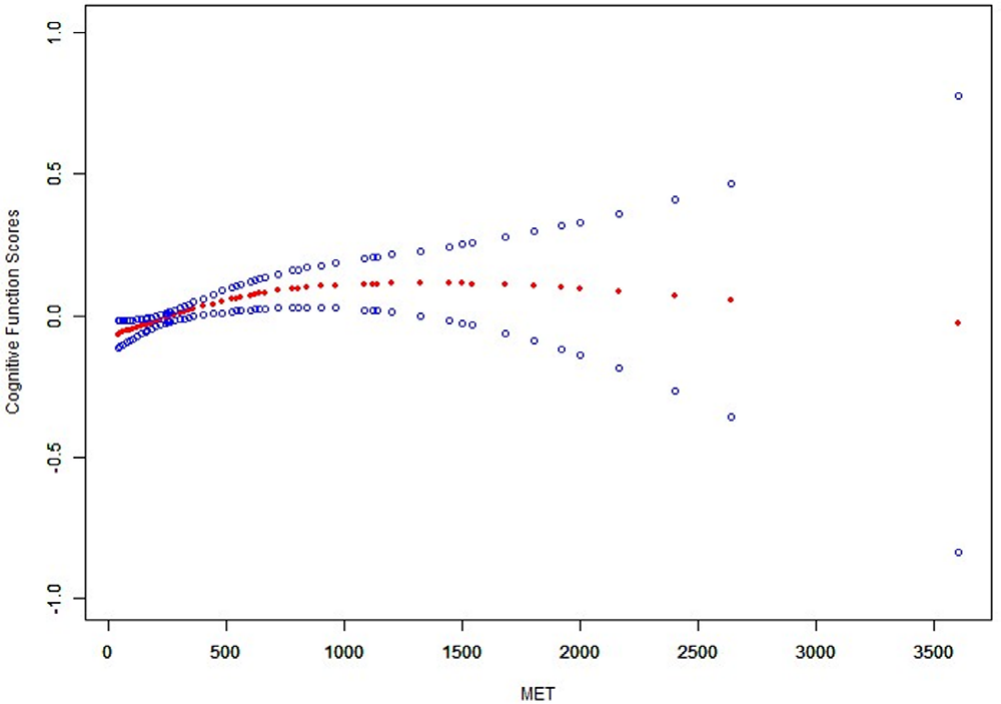
Association between MET and cognitive function with smoothed curve fitting. MET = metabolic equivalent.

Smoothed curve fitting (Fig. 5) further validated this relationship, demonstrating a steady increase in cognitive function scores with rising MET levels. The upward trend was particularly pronounced in the higher MET range. These findings underscore recreational physical activity as a promising intervention strategy for improving cognitive function in older adults.

### 3.5. Mediation effects of recreational physical activities between BMI and cognitive function

Mediation analyses (Table 5) demonstrated that recreational physical activity partially mediated the relationship between BMI and cognitive function. In the fully adjusted model (Model 3), the direct effect of BMI on cognitive function was −0.0431 (*P* = .0540), while the indirect effect mediated through recreational physical activity was significant (−0.0088, *P* = .0140), resulting in a total effect of −0.0519 (*P* = .0180). Recreational physical activity accounted for 16.95% of the total effect (*P* = .0320).

**Table 5:** Mediation effects of recreational physical activities between BMI and cognitive function scores.

	Direct effect	*P*	Indirect effect	*P*	Total effect	*P*	Proportion mediated (%)	*P*
MET								
Model 1	0.0084	.5740	−0.0116	.0120	−0.0032	.3900	3.5978	.3980
Model 2	−0.0413	.0400	−0.0086	.0180	−0.0499	.0120	17.3004	.0300
Model 3	−0.0431	.0540	−0.0088	.0140	−0.0519	.0180	16.9500	.0320

Model 1: unadjusted model.

Model 2: adjusted for age, sex, race, and ethnicity.

Model 3: further adjusted for smoking status in addition to Model 2.

BMI = body mass index, MET = metabolic equivalent.

In contrast, in the unadjusted model (Model 1), the indirect effect was significant (−0.0116, *P* = .0120), but neither the direct effect nor the total effect reached statistical significance (*P* = .5740 for the direct effect and *P* = .3900 for the total effect). After adjusting for age, sex, race, and smoking status in Model 2, the indirect effect became more pronounced (−0.0086, *P* = .0180), and the proportion of the total effect mediated by recreational physical activity increased to 17.30% (*P* = .0300).

These findings provide empirical support for the potential mechanisms linking BMI to cognitive function, suggesting that recreational physical activity partially explains this association. Furthermore, the results highlight the critical role of recreational physical activity as a potential intervention factor capable of mitigating the adverse effects of BMI on cognitive function by enhancing physical activity levels. This underscores the importance of promoting recreational physical activity in strategies aimed at preserving cognitive health in older adults.

## 4. Discussion

### 4.1. Main findings

By analyzing data from NHANES, this study systematically examined the relationships among BMI, recreational physical activity (MET), and cognitive function in older adults. The findings revealed a significant negative association between BMI and cognitive function scores, indicating that higher BMI levels were linked to lower cognitive function, particularly among individuals with elevated BMI. In contrast, recreational physical activity was positively associated with cognitive function, with higher levels of physical activity corresponding to better cognitive performance. Mediation analyses further suggested that recreational physical activity partially explained the association between BMI and cognitive function, with the mediating effect accounting for 16.95% of the total effect. These findings imply that the negative association between BMI and cognitive function may, in part, be driven by reduced levels of recreational physical activity, though the cross-sectional design limits causal interpretations.

These findings provide novel insights into the complex interplay between BMI, recreational physical activity, and cognitive function from multiple perspectives. They highlight the critical role of recreational physical activity in potentially mitigating the adverse associations between BMI and cognitive health. While this study enhances the understanding of factors influencing cognitive health in older populations, it primarily lays a scientific foundation for future research and intervention studies aimed at improving cognitive function.

The findings of this study align with previous research in several ways while also offering new insights. First, we confirmed that higher BMI is associated with lower cognitive function, consistent with prior studies. Previous research suggests that obesity and overweight may be linked to changes in brain structure and function through mechanisms such as chronic inflammation, insulin resistance, and vascular dysfunction.^[27–29]^ By highlighting that the association between elevated BMI and cognitive function is particularly pronounced in older adults, our findings reinforce this observed relationship. Although some previous studies have suggested a U-shaped association between BMI and cognitive outcomes, with both low and high BMI associated with poorer cognitive performance, we did not observe a clear U-shaped pattern in the present analysis. Instead, the inverse association was more evident at higher BMI levels.

Second, this study confirmed the positive association between recreational physical activity and cognitive function, consistent with existing literature. Extensive research suggests that physical activity is associated with enhanced cognitive health, potentially by promoting neuroplasticity, improving cerebral blood flow, and reducing inflammation.^[30,31]^ Our study provided more precise results by quantifying recreational physical activity using METs. These findings indicate that higher levels of physical activity are significantly associated with better cognitive function in older adults, highlighting the importance of promoting active lifestyles in aging populations.

Moreover, unlike earlier studies, this work systematically quantified the mediating role of recreational physical activity in the association between BMI and cognitive function through mediation analysis. Recreational physical activity accounted for 16.95% of the total effect, suggesting that it may partially explain the observed association between BMI and cognitive function. This novel finding addresses an important gap in understanding the interplay among BMI, physical activity, and cognitive health, offering insights for future research exploring potential intervention pathways. Compared with previous studies that predominantly focused on univariate analyses, the present study offers more comprehensive evidence by simultaneously examining the complex relationships between BMI, recreational physical activity, and cognitive function. This integrative approach not only enhances our understanding of the underlying mechanisms but also provides valuable insights for designing public health policies and interventions aimed at preserving cognitive health in older adults.

This study identified a negative association between BMI and cognitive function, with recreational physical activity playing a significant mediating role. Several potential mechanisms may explain these findings. First, elevated BMI may impair cognitive function through metabolic dysregulation and chronic inflammation.^[32]^ Obesity is strongly linked to insulin resistance, dyslipidemia, and chronic low-grade inflammation, all of which may accelerate cognitive decline by disrupting neuronal function and compromising cerebrovascular health.^[27]^ Additionally, obesity has been associated with structural brain changes, such as reduced gray matter volume and white matter lesions, further exacerbating the adverse effects of BMI on cognitive function.^[33]^ Second, recreational physical activity may exert protective effects on cognitive function through multiple pathways. Physical activity increases the expression of brain-derived neurotrophic factor, promoting neuroplasticity and neuronal renewal, particularly in the hippocampus.^[34]^ Furthermore, by improving cardiovascular health, enhancing cerebral blood flow, and reducing oxidative stress, physical activity may play a preventive role in the development of neurodegenerative diseases.^[31,35]^ These mechanisms collectively support the observed association between higher levels of physical activity and improved cognitive function.

Finally, the mediating role of recreational physical activity may reflect its ability to partially counteract the negative effects associated with BMI. Individuals with higher BMI often engage in lower levels of physical activity, limiting their ability to mitigate cognitive decline through metabolic and inflammatory regulation.^[36]^ Recreational physical activity not only improves obesity-related metabolic dysfunction but may also benefit cognitive health through positive psychological effects, such as enhanced mood and reduced stress.

In summary, the findings of this study likely result from a combination of direct cognitive impairment caused by BMI-induced metabolic dysregulation and chronic inflammation, and the indirect neuroprotective and metabolic regulatory effects of recreational physical activity. These results provide a theoretical framework for further mechanistic research and the design of targeted interventions to preserve cognitive health.

### 4.2. Strengths and limitations

This study offers several strengths while acknowledging certain limitations that warrant further exploration.

#### 4.2.1. Strengths

A major strength of this study lies in its use of NHANES, a nationally representative, large-scale dataset encompassing diverse demographic characteristics and comprehensive health indicators. This robust dataset ensures the broad applicability and generalizability of the findings. Additionally, the study employed multiple regression analyses combined with a mediation model, systematically evaluating the relationships among BMI, recreational physical activity, and cognitive function. By quantifying the mediating role of recreational physical activity, the study provides novel insights into the complex interplay between these factors.

#### 4.2.2. Limitations

Several limitations of this study must be considered. First, the inclusion of covariates significantly influenced the effect sizes. Adjusting for age, sex, race, and smoking status attenuated the observed association between BMI and cognitive function, potentially masking the direct effects of BMI. However, these adjustments were necessary to minimize confounding. The choice of covariates may still impact the interpretation of results, and future research should aim to identify the most critical confounders to ensure more precise modeling. Second, the cross-sectional design limits the ability to infer causality. While associations between BMI, cognitive function, and recreational physical activity were observed, it remains unclear whether increased BMI directly leads to cognitive decline or whether cognitive decline triggers a feedback loop of reduced physical activity and increased BMI. Longitudinal studies are essential to elucidate these dynamic relationships over time. Finally, the assessment of recreational physical activity was based on self-reported data, which may introduce recall bias or reporting inaccuracies. Although MET values were used as a widely accepted quantitative measure of physical activity, this approach does not fully capture the complexity of activity intensity, frequency, or type, which may have nuanced effects on cognitive function.

Despite these limitations, this study provides valuable theoretical and empirical insights into the relationships among BMI, recreational physical activity, and cognitive function, offering a solid foundation for future research and intervention design. Addressing these limitations through longitudinal and experimental studies will enhance our understanding of the underlying mechanisms and inform the development of targeted strategies to preserve cognitive health.

## 5. Conclusion

In this study of older adults from NHANES, higher BMI was associated with lower cognitive function, and recreational physical activity was positively associated with cognitive function. Recreational physical activity also partially mediated the association between BMI and cognitive function. These findings suggest that recreational physical activity may be 1 pathway linking BMI to cognitive function in older adults. Because of the cross-sectional design, causal relationships cannot be established. Further longitudinal studies are needed to confirm these associations.

## Author Contributions

**Conceptualization:** Zheng Zhang.

**Software:** Zheng Zhang.

**Data curation:** Juan Li.

**Methodology:** Juan Li.

**Writing – original draft:** Zheng Zhang.

**Writing – review & editing:** Juan Li.
